# 

**Published:** 1898-02

**Authors:** 


					﻿Vol. XVIII.
FEBRUARY, 1898.
NO. 2.
THE
pOMlEOPATHlC PHYSICIAN,
A Monthly Journal of Homoeopathic Materia Medica
and Clinical Medicine.
“ He it a freeman whom truth makes free, and all are slaves betide."
"Seek the Truth: eome whence it may, cost what it will."
KrtlV AND PUBLMHBD BY
WALTER M. JAMES, M. D.
PHILADELPHIA :
ISTo. 1231 LOCUST STREET.
LONDON I
ALFRED HEATH & CO., 8olb Agbnts fob Gbkat Britain,
114 Ebury Street, Eaton Square.
Yearly Subscription, $2.60, invariably in advance. Single iiiMikara? 26 etc.
Intend at Che Philadelphia Peet-OSee ae Seoond-Claea Mall Matter.
				

